# Spirituality and mental health

**DOI:** 10.4103/0019-5545.44742

**Published:** 2008

**Authors:** Abraham Verghese

**Affiliations:** Retired Professor of Psychiatry, Christian Medical College, Vellore, Anugraha, 444 C, CSG Street, Kamalakshipuram, Vellore - 632 002, India

## INTRODUCTION

All along, the majority position of Psychiatry has been that Psychiatry has nothing to do with religion and spirituality. Religious beliefs and practices have long been thought to have a pathological basis, and psychiatrists over a century have understood them in this light. Religion was considered as a symptom of mental illness. Jean Charcot and Sigmund Freud linked religion with neurosis. DSM3 portrayed religion negatively by suggesting that religious and spiritual experiences are examples of psychopathology. But recent research reports strongly suggest that to many patients, religion and spirituality are resources that help them to cope with the stresses in life, including those of their illness. Many psychiatrists now believe that religion and spirituality are important in the life of their patients. The importance of spirituality in mental health is now widely accepted. As John Turbott[1] puts it, rapprochement between religion and psychiatry is essential for psychiatric practice to be effective. The Royal College of Psychiatrists, London, has a special group on Psychiatry and Spirituality. The American College of Graduate Medical Education mandates in its special requirements for residency training in Psychiatry, that all programs must provide training in religious and spiritual factors that can influence mental health. The World Psychiatric Association recently established a section on psychiatry and religion. Lukoff *et al*.[2] proposed that the diagnostic entities of religious and psychospiritual problems should be incorporated in DSM4 which has been accepted. DSM4, V 62.89 includes three categories—normal religious and spiritual experiences; religious and spiritual problems leading to mental disturbances; and mental disturbances with a religious and spiritual context. I understand that the Indian Psychiatric Society has formed a task force on spirituality and mental health which is urging the Medical council of India to include taking the spiritual history as part of psychiatric evaluation. Even so the importance of religion and spirituality are not sufficiently recognized by the psychiatric community. Religion does not have a place in most of the psychiatry text books. Only very few psychiatrists make use of religion and spirituality in the therapeutic situation.

This paper makes an attempt to bring out the importance of spirituality in mental health.

## WHAT IS SPIRITUALITY?

Spirituality is a globally acknowledged concept. It involves belief and obedience to an all powerful force usually called God, who controls the universe and the destiny of man. It involves the ways in which people fulfill what they hold to be the purpose of their lives, a search for the meaning of life and a sense of connectedness to the universe. The universality of spirituality extends across creed and culture. At the same time, spirituality is very much personal and unique to each person. It is a sacred realm of human experience. Spirituality produces in man qualities such as love, honesty, patience, tolerance, compassion, a sense of detachment, faith, and hope. Of late, there are some reports which suggest that some areas of the brain, mainly the nondominant one, are involved in the appreciation and fulfillment of spiritual values and experiences.[3–5]

## SPIRITUALITY AND RELIGION

Religion is institutionized spirituality. Thus, there are several religions having different sets of beliefs, traditions, and doctrines. They have different types of community-based worship programs. Spirituality is the common factor in all these religions. It is possible that religions can lose their spirituality when they become institutions of oppression instead of agents of goodwill, peace and harmony. They can become divisive instead of unifying. History will tell us that this had happened from time to time. It has been said that more blood has been shed in the cause of religion than any other cause. The medieval holy wars of Europe; the religion-based terrorism and conflicts of modern times are examples. We must remember that the institutions of religion are supposed to help us to practice spirituality in our lives. They need periodical revivals to put spirituality in place.

## SPIRITUAL DIMENSION IS IMPORTANT IN MENTAL HEALTH

Mental health has two dimensions—absence of mental illness and presence of a well-adjusted personality that contributes effectively to the life of the community. Ability to take responsibility for one's own actions, flexibility, high frustration tolerance, acceptance of uncertainty, involvement in activities of social interest, courage to take risks, serenity to accept the things which we cannot change, courage to change the things which we can change, the wisdom to know the difference between the above, acceptance of handicaps, tempered self-control, harmonious relationships to self, others, including Nature and God, are the essential features of mental health. Spirituality is an important aspect of mental health. St. Augustine prayed “O God, thou created us in thy image and our hearts will be restless until they find their rest in Thee.” Though Sigmund Freud looked upon religion as an illusion and neurosis, Carl Jung considered the psyche as a carrier of truth, powerfully rooted in the unconscious mind. Religion is important, directly and indirectly, in the etiology, diagnosis, symptomatology, treatment and prognosis of psychiatric disturbances. Lack of spirituality can interfere with interpersonal relationships, which can contribute to the genesis of psychiatric disturbance. Psychiatric symptoms can have a religious content. For example, the loss of interest in religious activities is a common symptom of depression. Too much and distorted religious practices are common in schizophrenia. It is well recognized that some religious states and experiences are misdiagnosed as symptoms of psychiatric illness. Visions and possession states are examples. The spiritual background of the patient will help in the diagnosis of psychiatric disturbance. They are important in the treatment of psychiatric disturbance because spiritual matters can be profitably incorporated in psychotherapy. Spirituality is important in the prognosis of psychiatric conditions. In the spiritual perspective, a differentiation must be made between cure and healing. Cure is the removal of symptoms. Healing is the healing of the whole person. Adversity often produces maturity. Hence in psychotherapy, the patient must be helped to accept the handicap and transform the handicap to a life of usefulness.

## SOME SIGNIFICANT CLINICAL AND RESEARCH FINDINGS

Recent studies show that religious beliefs and practices are supportive to cope with stresses in life and are beneficial to mental health.

Thomas Ashby Wills,[6] Professor of Epidemiology and population health at Albert Einstein College of Medicine developed a scale that determines how important is religion to people. This was administered to 1182 children in New York. It was found that religiosity kept children from smoking, drinking and drug abuse by buffering the impact of life stresses. Gene H. Brody,[7] a research professor of child and family development at the University of Georgia, Athens, found that parents who were more involved in church activities were more likely to have harmonious marital relationships and better parenting skills. That in turn enhanced children's competence, self-regulation, psychosocial adjustment and school performance. Miller *et al*.[8] made a 10-year follow up study on depressed mothers and their offsprings and reported that maternal religiosity and mother-child concordance in religiosity were protective against depression in the offspring. They also reported that low level of religiosity was associated with substance abuse in the offsprings.[9] J. Scott Tonigan,[10] a research professor of psychiatry at the University of New Mexico, followed up 226 patients of alcohol dependence and reported that spirituality predicts behavior such as honesty and responsibility which in turn promoted alcohol abstinence. Wagner and King[11] conducted a study involving three groups—one group of patients who had psychotic illness, one group of formal care givers, and a third group of informal caregivers. The existential needs were the most important for the patient group, while the other groups considered material needs such as housing and work as more important. Neeleman and King[12] surveyed the psychiatric practices of 231 psychiatrists in London. 73% had no religious affiliation, 28% had belief in God, 61% believed that religion can protect against mental illness, and 48% asked patients about their religious practices. Baetz *et al*.[13] surveyed 1204 psychiatrists and 157 psychiatric patients in Canada. 54% of psychiatrists believed in God, 47% asked patients regarding their religious beliefs, and 55% consulted clergy for the management of patients. Among the patients, 71% believed in God, and 24% preferred psychiatrists who were religious. In an Australian survey, a large majority of patients with psychiatric illness wanted their therapists to be aware of their spiritual beliefs and needs and believed that their spiritual practices helped them to cope better.[14] Mathai and North[15] constructed a questionnaire, consisting of 5 questions and gave it to 70 parents of children attending child and adolescent mental health clinic. They reported that majority of the parents believed that spiritual concerns were important and that therapists should consider their spiritual beliefs in the management of the problems of the children. In USA, Curlin *et al.*[16] conducted a study of psychiatrists and compared them with physicians from other specialities in their religious affiliations and found that psychiatrists showed less religious affiliations. Several empirical studies on psychiatrists' religious characteristics have indicated that psychiatrists are significantly less religious than the general population, their patients and other physicians.[17],[18] In a 12-year follow up of all articles appearing in American Journal of Psychiatry and Archives of General Psychiatry, 72% of the religious commitment variables were beneficial to mental health; participation in religious services, social support, prayer and relationship with God were beneficial in 92% of citations.[19] Similar findings were reported in a review of the Journal of Family Practice.[20] In a British epidemiological study, church going and active religion were found to be protective to vulnerability for depression by Brown and Prudo.[21] In a detailed study on suicide in Netherlands, Kerkoff [quoted by Sims[22]] reported that there was a decline in suicidal rate, which was concurrent with a religious revival. A study on the factors in the course and outcome of schizophrenia was conducted in the Department of psychiatry, Christian Medical College, Vellore.. It was a collaborative study among three centers—Vellore, Madras and Lucknow. A two-year and five-year follow up showed that those patients who spent more time in religious activities tended to have a better prognosis.[23],[24] The above reports strongly suggest that religious beliefs and practices of psychiatric patients should be given importance. The sense of hope and spiritual support that patients get by discussing religious matters help them to cope better. They also suggest that the importance of religion and spirituality is not sufficiently recognized by the psychiatric community. Mental health workers must take it seriously since psychiatry cannot afford to ignore the importance of spirituality and religion in psychiatry. Sims[22] gives two case histories which drives home this fact. One is the case of Jim who suffered from Korsakov's psychosis. He was so deteriorated that he mistook his wife for a hat. In the ward, others considered him as desolate individual. But his behavior in the chapel was normal. In absolute concentration and attention, he would partake Holy Communion. He did not forget anything nor did he show any signs of Korsakov's psychosis. The other patient had chronic schizophrenia. He used to hear a voice commanding him to jump out of the window. His simple devout mother had taught him to resist the voice by praying to God. His mind was destroyed, but the capacity for spiritual life was present. Unfortunately, on the final occasion, he was too late to pray and he lost his life. Sims makes a comment, “It is unfortunate that we as psychiatrists can be so crass as to neglect this area of life which is clearly important to many of our patients.” Andresen,[25] in an editorial, has pointed out that our civilization's “loss of soul” may cause psychiatric symptoms such as depression, obsessions, addictions, and violence. She has suggested that it is the responsibility of psychiatrists to remind the medical fraternity the necessity of putting back the soul in medical ethics and the fact that spirituality is of vital importance for the mental health of people.

## WHAT CAN WE DO?

As pointed out earlier, spiritual values and religious practices are important in the lives of our patients. Many of their problems may centre round existential preoccupations. It is therefore important that we incorporate spirituality and religious practices in our treatment protocol. We must propagate the Bio-psycho-socio-spiritual model in our approach in psychiatry. Harold Koening,[26] in his paper Religion and Mental health: what should psychiatrists do?, has made some suggestions in this area.

1. Psychiatric history should be catered to the patients' spiritual orientation and religious practices. When we take psychiatric history, we usually ask for the denomination the patient belongs. We do not try to find out how the patient experiences religion. What does religion and spirituality mean to the patient. The psychiatric history should gather information about patient's religious background and experiences in the past and what role religion plays in coping with life stresses. Has patient had any past negative religious experiences? Has he got spiritual and social support from the congregation which he attends? How active is he in the religious congregation? Some religious beliefs can be in conflict with the proposed treatment. Some religious groups are against any type of treatment. Some religious conflicts and frustrations may be contributing to the present psychiatric problem. Sexual abuse by religious workers, traumatic events which turned the patient away from religious beliefs and activities, unanswered prayers, etc. are examples. There are some questionnaires that can be used to take a history of spirituality and religious experiences.[15,27,28] Discussion with the patient on spiritual matters and religious experiences will strengthen the therapeutic relationship. It can also lead to reversal effect of a personal growth of the therapist.
2. We should respect and support patients' religious beliefs if these help them to cope better or do not adversely affect their mental health. For example, if a patient says that his discipline of fasting and prayer helps him to cope better, then this has to be encouraged. We should also challenge the beliefs that can adversely affect mental health. This has to be done very tactfully. It is better to be neutral till we understand the patients and the issues involved well and a good therapeutic relationship is formed. Patients may wish to discuss with the therapist regarding their subjective experiences and existential needs. We should spend time in listening to them.
3. Partnership with the religious workers is an useful area. Leavy and King[29] in their paper, The devil in the detail: partnership between psychiatry and faith based organizations, brings out the importance of such a partnership. They have reported that in UK, the clergy continue to have a central role in several communities and the utility of their involvement in the care of people with mental health problems is increasing. They have argued the importance of examining the form and parameters of partnership between the mental health team and the faith-based communities. For this partnership to be effective, the mental health workers must be spiritually oriented and the religious workers must be better informed about mental health and illness. As referred to earlier, some religious experiences are often misdiagnosed as symptoms of mental illness and vice versa, some psychiatric symptoms are explained as spiritual experiences. According to Sims,[22] phenomena such as faith, prayer and magic can lend themselves to description and definition using Jasperian phenomenology which can lead to a clear differentiation of normal and pathological religious experiences. This emphasizes the importance of psycho-pastoral partnership. One example of such a partnership is the Bangalore psycho-pastoral association, which runs a very efficient half-way home for psychiatric patients. Recognizing the importance of this, the World Council of Churches has formed an Advisory group on mental health and faith communities, which has been active in the exploration of strategies for an effective partnership between mental health services and faith communities. There can be problems in such a partnership. The religious workers may be reluctant to get involved in secular programs, leaving their spiritual fortress. Some of them can have incorrect ideas about the causes of mental illness, which can interfere with the treatment program. Some others can be against medical treatment. Mental health workers can also be prejudiced against the patients' religious beliefs and practices. As referred to earlier, research findings suggest that majority of psychiatrists do not give importance to the spiritual and religious experiences of patients. According to Neelman and Persaud,[30] this may be due to the following factors. Psychiatrists are by and large less religious than other physicians; psychiatrists often come to know of spirituality through the pathological religious symptoms of patients, which make them prejudiced against spirituality; psychiatrists tend to have a biological approach to mental illness, which ignores spiritual dimension; and psychiatrists may think that religion and spirituality cause dependence and guilt feelings. All these can be minimized with dialogue and periodical orientation programs. As John Turbott[31] puts it, psychiatry as a whole can only benefit if the concepts and vocabulary of religion and spirituality are more widely known and discussed among its practitioners. It is also true that conflicts and problems arise more with religious experiences and not with spirituality
4. Praying with the patient is a controversial area. Many psychiatrists will argue that it is a dangerous ground upon which to tread. If at all it is done, it should be done only after a strong therapeutic relationship is established and only if the patient asks for it. Praying for the patient can be beneficial.
5. Research. Although there is substantial body of literature that describes the connection between mental health and spirituality, we must develop theoretical models to understand their relationship in practice. The statistical findings reported earlier were mainly the results of surveys. High-quality evidence-based research is required to make the clinical applications more objective and effective. There are ample opportunities to do research in this area. Phenomena such as meditation, religious conversion, faith, mystical experiences, near death experiences, and rebirth concepts are all unchartered territories. What are their relation to normal life and psychiatric illness? What are the neural mechanisms which influence spiritual experiences?
6. Treatment. If spirituality is related to mental health and if religious beliefs and experiences are important in the life of the psychiatric patient, it is only natural that we should include religious concepts in psychotherapy. For example, some Christian, Gita, Buddhist and Quran passages can be profitably used to help the patient to cope with life situation. The spiritual concepts are incorporated in the treatment program of Alcoholic Anonymous. Seven out of the 12 AA steps relate to spirituality.

D'Souza[32] describes a new psychotherapeutic method, which is called Spiritually Augmented Cognitive Behaviour Therapy (SACBT). This is a treatment technique, incorporating spiritual values to Cognitive behavior therapy, which was developed and promoted at the University of Sydney. Four key areas are emphasized—acceptance, hope, achieving meaning and purpose and forgiveness. The patient is guided through five phases to achieve meaning and purpose. This starts with examining the inevitables of life such as birth and death. After desensitizing the patient to mortality, the patient is moved to the next phase of letting go of fear and turmoil in life. The next phase examines the patient's lifestyle aspects that avoid confronting mortality and perpetuate fear and turmoil. The next phase involves a focus on seeking divine purpose, after examining and accepting one's journey in life. Finally, meaning is sought by seeking meaning for each day. This is achieved by identifying meaningful and realistic factors within whatever limitations life and illness bring. The main techniques are empathic listening, facilitation of emotional expression and problem solving. The use of meditation, prayers and rituals together with monitoring effects of beliefs and rituals on symptoms form the behavioral components of the treatment. When the patient shows negative cognition, cognitive restructuring is employed. Generally, the treatment takes about 16 sessions, each lasting about 1 hour. The main indications are depression and adolescent problems. Randomized controlled trials show that SACBT produces significant improvement.

## References

[1] Turbott J (1996). Religion, spirituality and psychiatry: conceptual, cultural and personal challenges. Aust N Z J Psychiatry.

[2] Lukoff D, Lu F, Turner R (1992). Toward a more culturally sensitive DSM-IV. Psychoreligious and psychospiritual problems. J Nerv Ment Dis.

[3] Abraham J (2004). The quest for the spiritual neurone. Religion and Science series, No 1.

[4] Timble M (2008). Soul in the brain: the cerebral basis of language,art and belief. BJP.

[5] Saver JL, Rabin J (1997). The neural substraits of religious experience. J Neuropsychiatry Clin Neurosci.

[6] Wills TA, Gibbons FX, Gerrard M, Murry VM, Brody GH (2003). Family communication and religiosity related to substance abuse and sexual behaviour in early adolescence: a test for pathways through self control prototype perceptions. Psychol Addict Behav.

[7] Brody GH (2003). Religiosity and family relationships. Journal of marriage and the family.

[8] Miller L, Warner V, Wickramaratne P, Weissman M (1997). Religiosity and depression: ten-year follow-up of depressed mothers and offspring. J Am Acad Child Adolesc Psychiatry.

[9] Miller L, Davies M, Greenwald S (2000). Religiosity and substance abuse among adolescents in the National Comorbidity Survey. J Am Acad Child Adolesc Psychiatry.

[10] Tonigan JS (2003). Project match treatment participation and outcome by self-reported ethnicity. Alcohol Clin Exp Res.

[11] Wagner LC, King M (2005). Existential needs of people with psychiatric disorders in Porto Aligre, Brazil. Br J Psychiatry.

[12] Neeleman J, King M (1993). Psychiatrists' religious attitudes in relation to their clinical practice: a survey of 231 patients. Acta Psychiatr Scand.

[13] Baetz M, Griffin R, Bowen R, Marcoux G (2004). Spirituality and psychiatry in Canada:psychiatric practice compared with patient satisfaction. Can J Psychiatry.

[14] D'Souza RF (2002). Do patients expect psychiatrists to be interested in spiritual values?. Australasian psychiatry.

[15] Mathai J, North A (2003). Spiritual history of parents of children attending a child and adolescent mental health service. Australasian psychiatry.

[16] Curlin FA, Lawrence RE, Odell S, Chin MH, Lantos JD, Koenig HG (2007). Religion, spirituality and medicine. Am J Psychiatry.

[17] Galanter M, Larson D, Rubenstone E (1991). Christian psychiatry: the impact of evangelical belief on clinical practice. Am J Psychiatry.

[18] Curlin FA, Lantos JD, Roach CJ, Sellergren SA, Chin MH (2005). Religious characteristics of US physicians: a national survey. J Gen Intern Med.

[19] Larson DB, Sherrill KA, Lyons JS, Craigie FC, Thielman SB, Greenwold MA (1992). Association between dimensions of religious commitment and mental health, reported in American J of Psychiatry and Archives of General Psychiatry: 1978-1989. Am J Psychiatry.

[20] Craigie FC, Larson DB, Liu IY (1990). References to religion in the Journal of Family Medicine: dimensions and valence of spirituality. J Fam Pract.

[21] Brown GW, Prudo R (1981). Psychiatric disorders in a rural and an urban Population:etiology of depression. Psychological Medicine.

[22] Sims A (1994). ‘Psyche’--spirit as well as mind?. Br J Psychiatry.

[23] Verghese A, John JK, Rajkumar S, Richard J, Sethi BB, Trivedi JK (1989). Factors associated with the course and outcome of schizophrenia: results of a two year follow up study. Br J Psychiatry.

[24] Verghese A, John JK, Rajkumar S, Richard J, Sethi BB, Trivedi JK (1990). Factors associated with the course and outcome of schizophrenia: results of a five year follow up study. Br J Psychiatry.

[25] Andreasen NC (1996). Body and soul Am J Psychiatry.

[26] Koening HG (2008). Religion and mental health: what shall psychiatrists do?. Psychiatry Bulletin.

[27] Vaishnava M (2006). Spirituality and Psychiatry—complementary or contradictory?. Archives of Indian Psychiatry.

[28] King M, Speck P, Thomas A (2001). Royalfree interview for religious and spiritual beliefs: development and validation of a selfreport version. Psychol Med.

[29] Leavey G, King M (2007). The devil in the detail: partnership between psychiatry and faith based organizations. Br J Psychiatry.

[30] Neeleman J, Persaud R (1995). Why do psychiatrists neglect religion?. Br J Med Psychol.

[31] Turbott J (2004). Religion, spirituality and psychiatry: steps towards rapproachment. Australas Psychiatry.

[32] D'Souza RF (2004). Spiritually augmented cognitive behaviour therapy[SACB]. Australas Psychiatry.

